# Characterization of the complete mitochondrial genome sequence of *Tanichthys albiventris* and phylogenetic analysis

**DOI:** 10.1080/23802359.2023.2171244

**Published:** 2023-02-02

**Authors:** Tiezhu Yang, Chenxi Tan, Wenhao Sun, Dongpu Li, Liangjie Zhao, Fan Li

**Affiliations:** a College of Fisheries, Xinyang Agriculture and Forestry University, Xinyang, China; b Fishery Biological Engineering Technology Research Center of Henan Province, Xinyang, China; c Branch of Shanghai Science and Technology Museum, Shanghai Natural History Museum, Shanghai, China; d Institute of Hydrobiology, The Key Laboratory of Aquatic Biodiversity and Conservation of Chinese Academy of Sciences, Chinese Academy of Sciences, Wuhan, China

**Keywords:** Tanichthyidae, *Tanichthys albiventris*, *Tanichthys albonubes*, mitochondrial genome, phylogenetic analysis

## Abstract

In this study, we sequenced, annotated, and characterized the mitogenome of *Tanichthys albiventris* for the first time. The complete mitogenome is 16,544 bp in length, consisting of 13 protein-coding genes (PCGs), 22 transfer RNA genes, and two ribosomal RNA genes. We found nine genetic overlaps and 17 intergenic spacer regions throughout the mitogenome of *T. albiventris*. The A + T content of the mitogenome is 60.93%. All PCGs have the same start codon of the standard ATG, excepting for that of cytochrome c oxidase 1 (*cox1*) which is the GTG. A phylogenetic analysis with another 15 species of the Cyprinidae was performed using MrBayes and IQtree, based on the amino acid sequences of 13 mitochondrial PCGs. The results indicated that *T. albiventris* shares a close ancestry with *Tanichthys albonubes*.

*Tanichthys* is a small freshwater fish belonging to the family Tanichthyidae within the superfamily Cyprinoidea (Chen and Mayden 2009; Mayden and Chen 2010; Stout et al. 2016). It has become a famous ornamental fish owing to its colorful appearance (Weitzman and Chan 1966). *Tanichthys* are distributed in several basins in southeastern China, and northern and central Vietnam (Lin 1932; Freyhof and Herder 2001; Thang et al. 2019; Li et al. 2022). The wild populations of *Tanichthys* have been classified as a second-class protected animal in China due to habitat loss (Yue and Chen 1998). To date, 12 valid species have been reported within the genus *Tanichthys* (Freyhof and Herder 2001; Thang et al. 2019; Jin et al. 2022; Li et al. 2022). *Tanichthys albiventris* Li, Bohlen & Liao, 2022 can be distinguished from all other congeners by the number of branched dorsal- and anal-fin rays, and the color of dorsal- and anal-fin margins (Li et al. 2022).

In this study, the complete mitochondrial genome of *T. albiventris* was sequenced and characterized. The samples of *T. albiventris* (Figure 1) were collected from the Jiangping River (E108.0811 and N21.6548) in Jiangping Town, Dongxing City, Guangxi Province, China, and stored in Xinyang Agricultural and Forestry University Aquatic Museum (https://www.xyafu.edu.cn/, Liangjie Zhao and a850924t@163.com), voucher number XYAFU-Mu-1804158. Prior to further processing, samples were placed in 100% ethanol during collection and stored at −80 °C before DNA extraction. Total DNA was extracted from caudal fin tissue using the Ezup Column Animal Genomic DNA Purification Kit (Sangon Biotech (Shanghai) Co., Ltd., Shanghai, China). The quality of the isolated DNA was detected by 1.2% agarose gel electrophoresis and the DNA was stored at −20 °C until utilization. Long and accurate PCR (LA PCR) was performed using a set of specific primers to amplify the complete mitochondrial genome sequence. Based on the sequenced and published sequences of other *Tanichthys* species in the NCBI database, nine pairs of specific primers were successfully screened in this study, and the amplified products ranged from 864 bp to 3829 bp, covering the entire length of the mitochondrial gene sequence. PCR products were sequenced at Shanghai Biotech using an ABI 3730XL DNA analyzer (Applied Biosystems Inc., Foster City, CA).

**Figure 1.**
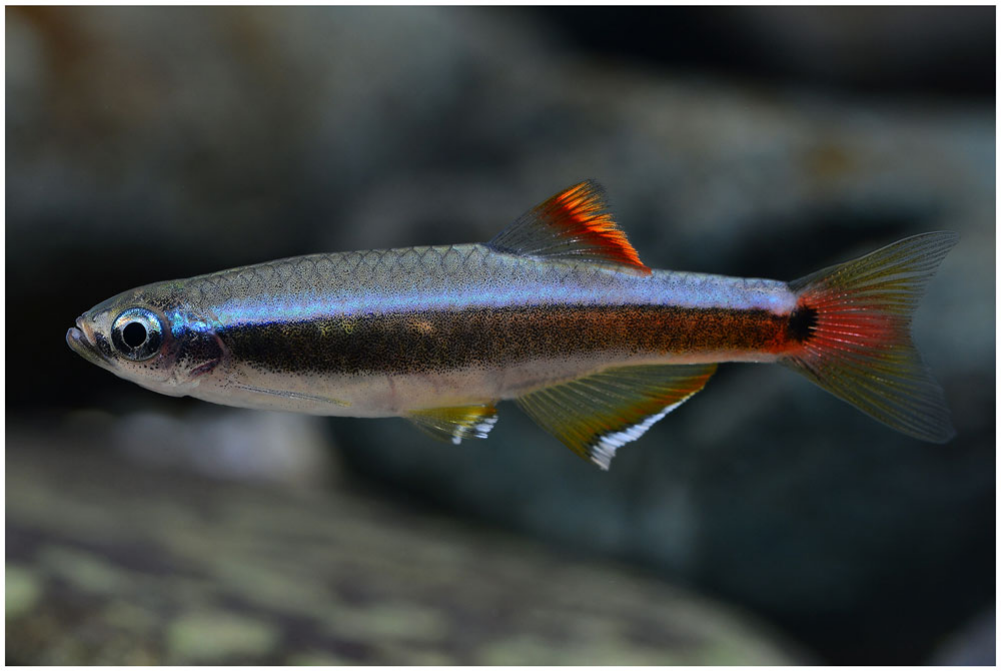
The morphological characteristics of *T. albiventris* (photographed by Fan Li).

The complete mitochondrial genome of *T. albiventris* is 16,544 bp in length and deposited in GenBank with the accession no. OM687295. It consists of 13 protein-coding genes (PCGs), 22 tRNA genes, and two rRNA genes (Figure 2). Twelve of the PCGs are located in the heavy strand, while only the gene encoding nad6 is located in the light strand. Similar to the preference of PCGs located in the heavy strand, 14 of the 22 tRNA genes are also located in the heavy strand. The ATG is commonly used as the start codon in this mitogenome with the exception of cytochrome c oxidase 1 (*cox1*) which starts with the GTG. Except for the incomplete stop codon of ‘T–’ in *cox2* and *cob* as well as that of ‘TA–’ in *cox3* and *nad4*, the remaining PCGs all stop with the TAG or TAA. The 22 tRNA genes were found to fold into a typical cloverleaf secondary structure by the analysis with the MITOS web server (Bernt et al. 2013). The overall basic nucleotide composition of the heavy strand in *T. albiventris* consists of 31.62% A, 29.31% T, 15.34% G, and 23.73% C, with an A + T content of 60.93%.

**Figure 2.**
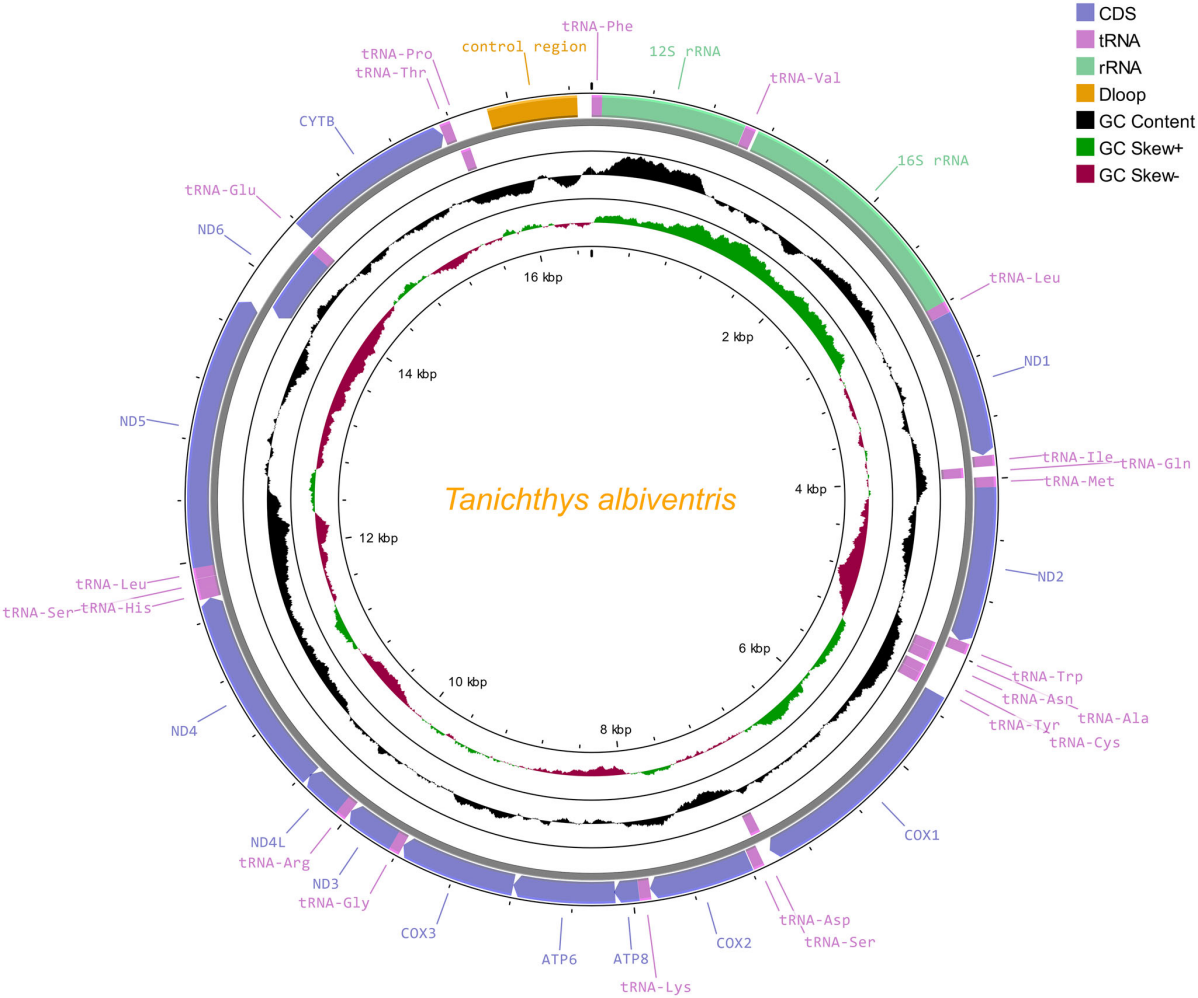
Circular map of the mitogenome of *T. albiventris.*

A total of 16 species (including this study species) from eight genera of Cyprinidae fishes were selected, and the protein sequences of the 13 PCGs were used to construct a phylogenetic evolutionary relationship tree. The mitogenome of *Cobitis lutheri* (accession no. AB860297) was chosen as the outgroup data (Maddison et al. 1984). The phylogenetic analysis was performed by MrBayes v3.2.6 and IQtree v1.6.10 (Ronquist et al. 2012; Minh et al. 2020). PartitionFinder 2.1.1 was employed to select the optimal evolutionary models for phylogenetic analysis (Lanfear et al. 2017). The resulting phylogenetic trees can be directly viewed in FigTree v1.4.2.

The constructed phylogenetic tree based on the optimal model is shown in Figure 3. The results indicated that *T. albiventris* is most closely related to *T. albonubes*, and they both clustered with *Tanichthys micagemmae* and *Tanichthys kuehnei*. The molecular phylogenetic analysis of the present study support the attribution of *T. albiventris* to the genus *Tanichthys* (Li et al. 2022), and the monophyly of the family Tanichthyidae (Mayden and Chen 2010; Stout et al. 2016), but disagree with the result of Tang et al. (2010) that the genera *Tanichthys* and *Tinca* constitute a monophyletic family. The comparative mitogenomic analysis of *T. albiventris* may provide valuable phylogenetic information.

**Figure 3.**
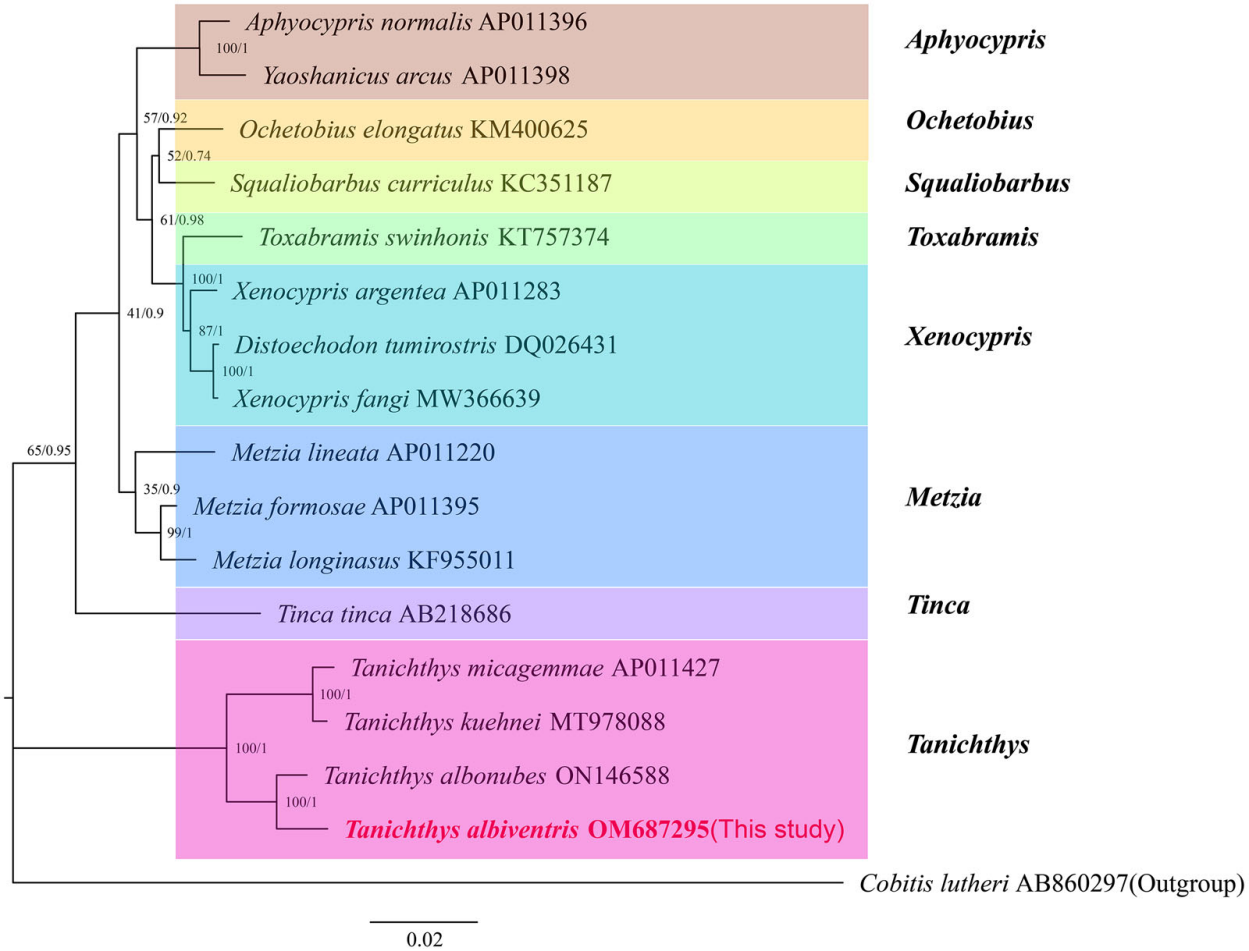
Phylogenetic tree constructed using the IQtree and MrBayes method based on the amino acid sequences of 13 proteins translated by the mitochondrial genome. The numbers on the branches indicate the posterior probabilities (IQtree/MrBayes). The red font represents the target species in this study.

## Data Availability

The genome sequence data that support the findings of this study are openly available in GenBank of NCBI at https://www.ncbi.nlm.nih.gov/ under the accession no. OM687295. The associated BioProject, SRA, and Bio-Sample numbers are PRJNA887552, SRR21820798, and SAMN31172242, respectively.

## References

[CIT0001] Bernt M, Donath A, Jühling F, Externbrink F, Florentz C, Fritzsch G, Pütz J, Middendorf M, Stadler PF. 2013. MITOS: improved de novo metazoan mitochondrial genome annotation. Mol Phylogenet Evol. 69(2):313–319. 22982435 10.1016/j.ympev.2012.08.023

[CIT0002] Chen WJ, Mayden RL. 2009. Molecular systematics of the Cyprinoidea (Teleostei: Cypriniformes), the world’s largest clade of freshwater fishes: further evidence from six nuclear genes. Mol Phylogenet Evol. 52(2):544–549. 19489125 10.1016/j.ympev.2009.01.006

[CIT0003] Freyhof J, Herder F. 2001. *Tanichthys micagemmae*, a new miniature cyprinid fish from central Vietnam (Cypriniformes: Cyprinidae). Ichthyol Explor Freshw. 12:215–220.

[CIT0004] Jin J, Li C, Zhao J. 2022. Descriptions of six new species in White Cloud Mountain minnow *Tanichthys albonubes* complex (Cypriniformes: Tanichthyidae) in southern China. J Fish Biol. 100(4):1062–1087. 35174507 10.1111/jfb.15012

[CIT0005] Lanfear R, Frandsen PB, Wright AM, Senfeld T, Calcott B. 2017. PartitionFinder 2: new methods for selecting partitioned models of evolution for molecular and morphological phylogenetic analyses. Mol Biol Evol. 34(3):772–773. 28013191 10.1093/molbev/msw260

[CIT0006] Li F, Liao T-Y, Bohlen J, Shen Z-X, Zhao L-J, Li S. 2022. Two new species of *Tanichthys* (Teleostei: Cypriniformes) from China. J Verteb Biol. 71(21067):1–13.

[CIT0007] Lin S. 1932. New cyprinid fishes from white cloud mountain, Canton. Lingnan Sci J. 11:379–383.

[CIT0008] Maddison WP, Donoghue MJ, Maddison DR. 1984. Outgroup analysis and parsimony. Syst Biol. 33(1):83–103.

[CIT0009] Mayden RL, Chen WJ. 2010. The world’s smallest vertebrate species of the genus *Paedocypris*: a new family of freshwater fishes and the sister group to the world’s most diverse clade of freshwater fishes (Teleostei: Cypriniformes). Mol Phylogenet Evol. 57(1):152–175. 20398777 10.1016/j.ympev.2010.04.008

[CIT0010] Minh BQ, Schmidt HA, Chernomor O, Schrempf D, Woodhams MD, von Haeseler A, Lanfear R. 2020. IQ-TREE 2: new models and efficient methods for phylogenetic inference in the genomic era. Mol Biol Evol. 37(5):1530–1534. 32011700 10.1093/molbev/msaa015 PMC7182206

[CIT0011] Ronquist F, Teslenko M, van der Mark P, Ayres DL, Darling A, Höhna S, Larget B, Liu L, Suchard MA, Huelsenbeck JP, et al. 2012. MrBayes 3.2: efficient Bayesian phylogenetic inference and model choice across a large model space. Syst Biol. 61(3):539–542. 22357727 10.1093/sysbio/sys029 PMC3329765

[CIT0012] Stout CC, Tan M, Lemmon AR, Lemmon EM, Armbruster JW. 2016. Resolving Cypriniformes relationships using an anchored enrichment approach. BMC Evol Biol. 16(1):244. 27829363 10.1186/s12862-016-0819-5 PMC5103605

[CIT0013] Tang KL, Agnew MK, Hirt MV, Sado T, Schneider LM, Freyhof J, Sulaiman Z, Swartz E, Vidthayanon C, Miya M, et al. 2010. Systematics of the subfamily Danioninae (Teleostei: Cypriniformes: Cyprinidae). Mol Phylogenet Evol. 57(1):189–214. 20553898 10.1016/j.ympev.2010.05.021

[CIT0014] Thang HN, Bohlen J, Dvorák T, Šlechtová V. 2019. *Tanichthys kuehnei*, new species, from Central Vietnam (Cypriniformes: Cyprinidae). Ichthyol Explor Freshw. 1081:1–10.

[CIT0015] Weitzman SH, Chan LL. 1966. Identification and relationships of *Tanichthys albonubes* and *Aphyocypris pooni*, two cyprinid fishes from South China and Hong Kong. Copeia. 1966(2):285–296.

[CIT0016] Yue PQ, Chen YY. 1998. China red data book of endangered animals: Pisces. Beijing: Science Press.

